# Risky sexual behaviours among young people in sub-Saharan Africa: how can parents use the Ottawa Charter for Health Promotion for change?

**DOI:** 10.1080/17290376.2019.1636710

**Published:** 2019-07-04

**Authors:** Elvis Tarkang, Lilian Pencille, Hubert Amu, Joyce Komesour, Prosper Lutala

**Affiliations:** a Department of Population and Behavioural Sciences, University of Health and Allied Sciences, Ho, Ghana; b HIV/AIDS prevention research network Cameroon (HIVPREC), Kumba, Cameroon; c Department of Family Medicine, College of Medicine, University of Malawi, Blantyre, Malawi

**Keywords:** Risky sexual behaviours, Ottawa Charter for Health Promotion, Sub-Saharan Africa, yo ung people, Health promotion, Ottawa charter, youths, behaviour change

## Abstract

Despite progress made in the treatment of HIV and AIDS by making available the antiretroviral treatment in Africa, youth are still struggling with inadequate knowledge, a negative attitude towards AIDS and high-risk sexual behaviour. All of these are compounded by a lack of open communication between parents and youths which among others, which impedes on the control of the pandemic in this vulnerable group. Building on ‘Ottawa Charter for Health Promotion’ as one way of breaking all barriers in this communication, we suggest keys points in five different domains of this framework namely: building healthy public policies, creating a supportive environment, strengthening community action, developing personal skills, and reorienting the health services.

## Introduction

HIV/AIDS remains a global health challenge with an estimated 35 million people living with the disease globally (WHO, n.d.), and 71% residing in sub-Saharan Africa (SSA) (Unicef Data, 2019). This sub-region also accounts for about 70% of new infections and 74% of HIV-related deaths (Unicef Data, 2019). Young people aged 15–24 years account for over 40% of new HIV infections globally (UNAIDS, n.d.), and about 50% in SSA (UNAIDS, 2004, 2011), due to inadequate knowledge and negative attitudes regarding the disease, as well as risky sexual behaviours (Chen, 2008; Irwin et al., 1997; Hall, Holmqvist, & Sherry, 2004). Monitoring the sexual behaviours of this vulnerable age group is, therefore, necessary in order to control the HIV/AIDS pandemic in SSA (Irwin et al., 1997; Hall, Holmqvist, & Sherry, 2004).

In SSA, the HIV pandemic is primarily driven by high-risk heterosexual practices which account for about 90% of the infection (Chen, 2008). Young people are particularly vulnerable to HIV infection because of the risky sexual behaviours they engage in. These behaviours are influenced by lack of access to accurate and personalised HIV information and prevention services, socio-economic reasons, lack of parental control, peer pressure and lack of youth-friendly recreational facilities (Agardh, Cantor-Graae, & Östergren, 2012).

Sex education has rarely been a comfortable topic for parent–child communication especially in SSA (Agardh et al., 2012). Many parents are either unwilling to talk about sex, are uncomfortable doing so, or lack the knowledge themselves (UNICEF, 2002b). Many barriers prevent open communication between parents and children about sexual issues. Adults, for instance, fear that informing young people about sex and teaching them how to protect themselves will make them sexually active (UNICEF, 2002b). Parents, therefore, play passive roles in providing information regarding sexual education to their children. This is because sex in most SSA societies is a taboo subject between parents and children. In urban areas, most parents are workers and spend little time with their children. Children, therefore, spend their time with grandparents or other persons entrusted with their care, yet communication about sex and sexuality are a silent wave (UNICEF, 2002a).

Since there is no cure or vaccine for HIV/AIDS, prevention of new infections must be the cornerstone for combating HIV/AIDS. Moreover, preventive measures through educating young people are the only ‘vaccines’ available for this disease (UNAIDS, 2012).

The former president of South Africa, Nelson Mandela, emphasised that ‘good morals and socially accepted conduct must be melded within families, and that, good parents are responsible for doing so’ (Nefale, 2001). Children subject to an authoritarian parenting style are more obedient and conform well to standards set by their parents (Moen, Elder, & Luscher, 1995). As such, the family must be at the nucleus of the struggle against the HIV/AIDS pandemic (Nefale, 2001).

Nevertheless, parents fail to fulfil their role of educating their children on sex, sexuality and HIV/AIDS (The Joint United Nations Program on HIV/AIDS/Economic Commission for Africa. AIDS in Africa: country by country, 2000). This is reflected in the high prevalence of HIV/AIDS among young people aged 15–24 in SSA (UNAIDS, 2004, 2011). Young people find it difficult to obtain precise information on HIV/AIDS from their parents because the parents are either ignorant or misinformed of their parenting role (Wilbraham, 2002). Parents might also be oblivious of the risks involved if they do not educate their children on sexual matters; they may also lack the ability to communicate with their children on HIV/AIDS and sexuality. This causes parents to be reticent about the knowledge they have concerning HIV/AIDS and thus, unable to transfer it to their children, leading to role ambiguity. Some parents may be knowledgeable about HIV and AIDS but are overpowered by their children. Therefore, they fail to transfer their knowledge to them.

Individuals form their own views in consonance with or in opposition to the dominant norms of their peers, family and society (Meekers, Klein, & Foyet, 2003). Family support is, therefore, critical, given the strong influences that the family environment exerts on adolescent sexuality especially in SSA (Meekers et al., 2003).

Risky sexual behaviour among young people in SSA is a major public health problem. Young people aged 15–24 years comprise approxima tely half of all new HIV cases in SSA (UNAIDS, 2004, 2011), the majority of whom are infected through unprotected sex (Centers for Disease Control and Prevention, 2005; UNAIDS, 2004). Condom use is effective in preventing HIV infection and some family-based prevention programmes hope to increase safer sex by improving parent–adolescent communication (Pequegnat, 2000).

When parents talk with their children about sex, they report greater safe sexual practices (DiClemente et al., 2001; Romer et al., 1999). For example, mother–child discussions about condom use prior to sexual debut have been correlated with adolescent condom use at last sex (Miller, Levin, Whitaker, & Xu, 1998). Similarly, 76% of sexually active adolescents, who reported having had a conversation with either parent about condoms, used a condom at most recent sexual intercourse and also reported greater lifetime condom use than those who had not discussed condoms with their parents (Whitaker & Miller, 2000). African parents thus have a critical role in influencing the attitudes and beliefs of young people toward sexual risk behaviours. However, parents need information, skills and community support to assist them in guiding their adolescents’ safe sexual practices.

## Ottawa Charter for Health Promotion

The Ottawa Charter for Health Promotion came out of the first International Conference on Health Promotion organised by the World Health Organization (WHO), which was held in Ottawa, Canada on 21 November, 1986 (WHO, 1986). The conference was organised as part of efforts aimed at promoting action towards the achievement of ‘Health for All’ by the year 2000 and beyond. Health promotion was defined as ‘is the process of enabling people to increase control over, and to improve their physical, mental and social wellbeing’ (WHO, 1986).

According to the Charter, to reach a state of complete physical, mental and social well-being, an individual or group must be able to identify and to realise aspirations, to satisfy needs, and to change or cope with the environment. Health is, therefore, seen as a resource for everyday life, not the objective of living. Health is a positive concept emphasising social and personal resources, as well as physical capacities. Therefore, health promotion is not just the responsibility of the health sector but goes beyond healthy life-styles to well-being. The charter posits that the fundamental conditions and resources for health are peace, shelter, education, food, income, a stable eco-system, sustainable resources, social justice, and equity (WHO, 1986).

There are five key action areas of the Ottawa Charter for health promotion which provide strategies from which governments and health promoters in SSA can support parents to promote health and encourage safe sexual practices among young people. These are building healthy public policy, creating a supportive environment, strengthening community action, developing personal skills, and re-orienting the health services (WHO, 1986).

### Building healthy public policies

Health promotion policy combines diverse but complementary approaches including legislation, fiscal measures, taxation and organisational change (WHO, 1986). It is a coordinated action that leads to health, income and social policies that foster greater equity. The aim must be to make the healthier choice the easier choice for the population and policy-makers as well (WHO, 1986).

In the area of sexual risk behaviours among young people in SSA, governments should include in their national action plans on HIV/AIDS, a commitment to educate parents on the pandemic and create a supportive environment to facilitate parent-youth communication regarding HIV/AIDS and sexuality. Also, policies should be developed to increase the self-efficacy of parents as this has the propensity of enabling them to successfully impart HIV/AIDS-related knowledge to their children at home.

### Creating a supportive environment

A supportive environment is essential for health (Murdoch-Kinch et al., 2017). Supportive environments cover the physical, social, economic, and political environment. Supportive environments encompass where people live, work and play (WHO, 1986). Supportive environments are ones that provide young people protection from threats to their health, resilience, and overall development (YouThrive, 2018). Such environments enhance their access to the supports, services, and other resources that foster resilience and promote health and well-being.

In the area of creating a supportive environment for safe sexual practices among youths, governments and other stakeholders should institute laws and legislations against cultural practices that predispose young people to HIV transmission, laws against sexual violence and rape, and strategies aimed at reducing poverty and unemployment as well as promoting gender equity. Parents should be at the forefront of all of these.

### Strengthening community action

According to the Ottawa Charter, health promotion works through concrete and effective community action in setting priorities, making decisions, planning strategies and implementing them to achieve better health (WHO, 1986).

Parents should be encouraged to form self-help groups in order to support one another in the area of parent-young person communication regarding sexuality and sexual behaviours and to care for parents infected or affected by the HIV/AIDS pandemic. The governments and other stakeholders in SSA should also train parents as community health workers and home-based care providers who will provide young people with the enabling environment to practice safe sex. Also, civil society and community service groups should carry out advocacy regarding training for parents on parent–adolescent communication regarding sexuality and on how to curb risky sexual behaviours among people.

### Developing personal skills

Health promotion supports personal and social development through providing information, education for health and enhancing life skills (WHO, 1986). By so doing, it increases the options available to people to exercise more control over their own health and over their environments, and to make choices conducive to health (WHO, 1986).

Strategies for empowering parents include leadership training, learning opportunities for health, and access to resources including materials and funding. Developing personal skills will help parents to identify the needs and concerns of their children regarding sexuality and sexual behaviour, and gain the power, skills and confidence to act upon them.

Life skills programmes should be part of the curriculum for parents aimed at promoting safe sexual behaviours for young people. There should also be a training of parents on parent–adolescent communication and on sexual risk behaviours and HIV/AIDS-related issues. Also, the mass media should be used to increase awareness of parents on risky sexual behaviours among young people, HIV/AIDS issues, and parent-young person communication.

### Reorienting the health services

According to the Ottawa Charter, the responsibility for health promotion in health services is shared among individuals, community groups, health professionals, health service institutions, and governments(WHO, 1986). The role of the health sector must, therefore, move increasingly in a health promotion direction, beyond its primary responsibility of providing clinical and curative services. Health services thus need to embrace an expanded mandate which is sensitive and respects cultural needs (WHO, 1986).

Governments of SSA countries should shift focus from curing sexually transmitted infections among young people to health promotion and prevention by empowering parents with the capacity and ability to handle sexual issues of young people and risky sexual behaviours among them. Also, more health promoters should be trained to empower parents with the skills regarding parent-young person communication on sexuality. Mobile support services for parents regarding parent-young person communication on sexuality and sexual behaviours should also be given priority in SSA countries.

## Conclusion

Programmes targeting these five areas of the Ottawa Charter for health promotion might enhance the knowledge and self-efficacy of parents in SSA thereby empowering them for adequate and fruitful discussions with their adolescent children regarding sexuality and sexual behaviour. This may go a long way to reduce risky sexual behaviours and in turn, curb the spread of HIV/AIDS among them. Full compliance with the five action areas of the Ottawa Charter for health promotion (WHO, 1986) to address risky sexual behaviours among young people in SSA, could go a long way in eliminating AIDS by 2030 and in achieving goal 3 of the UN sustainable development goals (to ensure healthy lives and promote well-being for all at all ages) (UNDP, 2015). AIDS still remains the leading cause of death for youths in SSA (UNAIDS, 2004, 2011), and risky sexual behaviours predispose them to the risk of acquiring HIV/AIDS (Irwin et al., 1997). One promising future direction for research could be in testing the acceptability, usability, feasibility, efficacy, effectiveness, and/or cost-effectiveness of implementing these key action areas in HIV among others, in an area in Africa.
